# OVERVIEW OF NIA BUDGET

**DOI:** 10.1093/geroni/igad104.0832

**Published:** 2023-12-21

## Abstract

Dr. Hodes will discuss the NIA budget, overall research priorities of the Institute, and key programs and initiatives.

